# Autoimmunity to phosphatidylserine and anemia in African Trypanosome infections

**DOI:** 10.1371/journal.pntd.0009814

**Published:** 2021-09-29

**Authors:** Juan Rivera-Correa, Joseph Verdi, Julian Sherman, Jeremy M. Sternberg, Jayne Raper, Ana Rodriguez

**Affiliations:** 1 Department of Microbiology, New York University School of Medicine, New York, United States of America; 2 Department of Biological Sciences, Hunter College of City University of New York, New York, United States of America; 3 School of Biological Sciences, University of Aberdeen, Aberdeen, United Kingdom; Liverpool School of Tropical Medicine, UNITED KINGDOM

## Abstract

Anemia caused by trypanosome infection is poorly understood. Autoimmunity during *Trypanosoma brucei* infection was proposed to have a role during anemia, but the mechanisms involved during this pathology have not been elucidated. In mouse models and human patients infected with malaria parasites, atypical B-cells promote anemia through the secretion of autoimmune anti-phosphatidylserine (anti-PS) antibodies that bind to uninfected erythrocytes and facilitate their clearance. Using mouse models of two trypanosome infections, *Trypanosoma brucei* and *Trypanosoma cruzi*, we assessed levels of autoantibodies and anemia. Our results indicate that acute *T*. *brucei* infection, but not *T*. *cruzi*, leads to early increased levels of plasma autoantibodies against different auto antigens tested (PS, DNA and erythrocyte lysate) and expansion of atypical B cells (ABCs) that secrete these autoantibodies. *In vitro* studies confirmed that a lysate of *T*. *brucei*, but not *T*. *cruzi*, could directly promote the expansion of these ABCs. PS exposure on erythrocyte plasma membrane seems to be an important contributor to anemia by delaying erythrocyte recovery since treatment with an agent that prevents binding to it (Annexin V) ameliorated anemia in *T*. *brucei*-infected mice. Analysis of the plasma of patients with human African trypanosomiasis (HAT) revealed high levels of anti-PS antibodies that correlated with anemia. Altogether these results suggest a relation between autoimmunity against PS and anemia in both mice and patients infected with *T*. *brucei*.

## Author summary

African trypanosomes are relevant pathogens and a cause of great morbidity and economic burden in endemic countries. Anemia during infection by African Trypanosomes is one of the most common pathologies associated with the disease, but the mechanisms leading to it are poorly understood. Here, we report evidence of autoimmunity, particularly autoantibodies against the lipid phosphatidylserine that are secreted by autoimmune/atypical B-cells, in *Trypanosoma brucei* infected mice and that are also found in Human African Trypanosomiasis patients and correlate with levels of anemia. These findings point to a relation between autoimmunity and anemia during infection with African Trypanosomes, possibly through the secretion of autoantibodies against phosphatidylserine.

## Introduction

Anemia is a very common, but poorly understood, complication in many infectious diseases, including protozoan parasitic infections, such as trypanosomiasis and malaria. Trypanosomes are eukaryotic parasites that cause a range of diseases and are characterized by their geographical endemicity. The most relevant human trypanosome pathogens are *Trypanosoma brucei* and *Trypanosoma cruzi*, that cause sleeping sickness in Africa, and Chagas disease in the Americas, respectively [1,2]. Both infections, but particularly *Trypanosoma brucei*, lead to some degree of anemia [3,4].

Autoimmunity is defined as a misguided response against the host own tissues, cells and molecules leading to pathology. Autoimmune anemia has been a well-documented complication during different infections but has been complex to understand due to its multi-factorial etiology [5]. During malaria, autoimmune antibodies targeting the membrane lipid phosphatidylserine (PS) exposed in the membrane of uninfected erythrocytes promote their lysis and premature clearance in both mice and humans, aggravating anemia [6–11]. These anti-PS autoantibodies are secreted primarily by autoimmune B-cells (called atypical B-cells or Age-Associated B-cells, ABCs), that are characterized by the expression of the transcription factor T-bet and the integrin CD11c, and associated with anemia during malaria [12]. A role for autoimmunity in promoting anemia during trypanosomiasis has been suggested [3] but remains poorly understood.

Using an acute mouse model of African trypanosomiasis, we show here that, similarly to malaria, there are early high levels of autoimmune antibodies in an acute *Trypanosoma brucei brucei (T*. *b*. *brucei)* mouse infection. This was not observed in an acute mouse model of Chagas disease. Our results also show an increase in the expansion of ABCs during acute *T*. *b*. *brucei* infection in mice, which are able to secrete specific anti-PS autoantibodies. We observed that *T*. *b*. *brucei* lysates could directly promote the differentiation of ABCs in synergy with other inflammatory signals, as described in other infections and autoimmune mice models. PS exposed on the surface of erythrocytes seems to be a significant target during autoimmune anemia, since injection of the specific PS-binding protein annexin V enhanced RBC recovery in *T*. *b*. *brucei*-infected mice. Lastly, levels of IgM and IgG anti-PS antibodies are significantly elevated in the plasma of Human African Trypanosomiasis (HAT) patients and correlate with anemia. Taken together, these results point to a relation between autoimmunity and *T*. *brucei*-induced anemia.

## Experimental procedures

### Ethics statement

The patient samples included in this manuscript are from a previous study [13] approved by the Grampian Research Ethics Committee (Aberdeen, UK) and the Ministry of Health (Uganda). This study was conducted according to the principles expressed in the Declaration of Helsinki. All patients recruited received written and verbal information explaining the purpose of this study and gave informed consent. The ethical committees in Uganda (Ministry of Health), Malawi (College of Medicine) and the UK (Grampian Joint Ethics Committee) approved all protocols. Ethical consent forms were designed in English and also translated into local languages. Consent was given as a signature or a thumb print after verbal explanation.

### Mice, parasites, and infections

This study was carried out in strict accordance with the recommendations in the Guide for the Care and Use of Laboratory Animals of the National Institutes of Health. The protocol was approved by the Institutional Animal Care and Use Committee of New York University School of Medicine, which are fully accredited by the Association For Assessment and Accreditation Of Laboratory Animal Care International (AAALAC). Female BALB/c and Swiss-Webster mice 6 to 8 weeks old were purchased from the National Institutes of Health (Bethesda, MD) and The Jackson Laboratory (Bar Harbor, ME). All protocols used have been validated and are routinely used by the NYU Anti-Infectives Screening Core.

For *T*. *brucei* infection, 6 Swiss-Webster mice were injected i.p. with 10^4^ *T*.*b*. *brucei* AnTaR 1 expressing *Renilla* luciferase [14] bloodstream forms obtained from culture in HMI-9 medium, resuspended in PBS, final volume 250 μl. The transgenic luciferase-expressing parasite was generously provided by Nick Van Reet, PhD, of the Institute of Tropical Medicine in Antwerp, Belgium [15]. Mice were kept until day 13 post-infection as indicated in our IACUC protocol.

For *T*. *cruzi* infection, 6 BALB/c mice were injected i.p. with 10^6^ trypomastigotes of *T*. *cruzi* Brazil strain expressing firefly luciferase [16] obtained from culture in NIH-3T3 cells. For purification, infected NIH-3T3 culture media was centrifuged for 7 min at 2500 rpm and trypomastigotes were allowed to swim out of the pellet for 3 h at 37°C. Supernatant containing free swimming trypanosomes was removed, centrifuged again and resuspended in PBS for injection at a final volume 250 μl per mouse. The luciferase-expressing parasite was generously provided by Barbara Burleigh, PhD, of Harvard University T. H. Chan School of Public Health, Boston, US.

At days 8 and 12 after infection, the mice were anesthetized by inhalation of isofluorane (controlled flow of 1.5% isoflurane in air was administered through a nose cone via a gas anesthesia system). Mice were injected with 200 μl of 150 mg/kg of D-Luciferin Potassium-salt (Goldbio) dissolved in PBS for *T*. *cruzi* infections and with 100 μl of a coelenterazine native solution (10 mg/mouse) for *T*. *brucei* and imaged 5 to 10 min after with an IVIS Lumina II imager (Xenogen, Alameda, CA). Data acquisition and analysis were performed with the software LivingImage (Xenogen). The luciferin signal is proportional to the parasite load.

For the in *vivo* model of anemia, mice were i.p. infected with *T*. *b*. *brucei* followed by i.v. injection of annexin V (200 μg/mouse, Sigma, St. Louis, MO) or vehicle (PBS) on days 3 and 7 post infection as established in a previous malarial anemia study [10]. Anemia was assessed by tail puncture blood collection and erythrocytes were counted in a hemocytometer. This experiment was performed in two independent infections, each with 6 mice total (3 mice per group). Values from 6 mice total were averaged for each time point.

For *Plasmodium yoelii* infections, female Swiss Webster mice were injected i.p. with 10^6^ infected RBCs per mouse of the nonlethal strain *P*. *yoelii* 17XNL resuspended in PBS, final volume 250 μl. To evaluate parasitemia, thin blood smears were made by bleeding mice from a nick in the tail. Smears were stained with KaryoMAX Giemsa (Life Technologies, Norwalk, CT), and a minimum of 500 RBCs per smear were counted. To evaluate anemia, RBC numbers were counted in Neubauer Chamber in an inverted light- microscope. *P*. *yoelii* 17XNL-infected RBCs were harvested by cardiac puncture of infected, anesthetized mice before the peak of parasitemia. RBCs were washed twice with PBS and separated from white blood cells by centrifugation at 2000 × g for 3 min. *P*. *yoelii*-infected RBCs were isolated through LD magnetic columns (Miltenyi) and infected erythrocytes were lysed by freeze–thaw 10 times with liquid nitrogen and kept sterile.

### Flow cytometry

All flow cytometry was performed on a FACSCalibur (Becton Dickinson, Franklin Lakes, NJ) and analyzed with FlowJo software (Tree Star, Ashland, OR). All samples were incubated with FcR-blocking agent (BD Bioscience, clone 2.4G2) prior to staining with fluorescent antibodies. To characterize mouse CD11c^+^T-bet^+^B cells the following antibodies (BioLegend (San Diego, CA) were used at 1 μg/ml: anti-CD19-FITC(6D5), anti-T-bet-PE (4B10), anti-B220-PRCP(RA3-6B2); and anti-CD11c-APC (N418). All antibodies were matched with their correct fluorescent-conjugated isotype control. Intracellular T-bet staining was performed using the True-Nuclear Transcription Factor Buffer Set (Biolegend) and following manufacturer’s instructions. In brief, for intracellular staining, cells were fixed after surface staining at 4°C with True-NuclearTranscription Factor 1X Fix reagent (Biolegend) for 40 minutes. Then cells were permeabilized with a Transcription Factor Staining Kit 1X Perm reagent by washing 3 times, followed by staining with anti-T-bet-PE (4B10) for 30 minutes, washed 3 more times with 1X Perm reagent and then resuspended in FACS buffer (PBS 1X 0.5% BSA 2mM EDTA) for flow cytometry analysis. To assess ABCs, we used standard ABC gating strategies [17–19] (S1 Fig).

To assess PS exposure on RBCs, Annexin V-FITC (Biolegend) diluted in annexin V binding buffer (BD Biosciences, San Jose, CA) was used following manufacturer’s protocol.

### ELISA

Costar 3590 96-well ELISA plates were coated with 100μL PS at 20 μg/ml, uninfected erythrocyte lysate (10^9^ erythrocytes/ml in PBS), *T*. *b*. *brucei* bloodstream forms or *T*. *cruzi* trypomastigotes lysates *(*10^6^ parasites/μl in PBS) diluted 1:500 in PBS, Calf thymus DNA (Sigma) diluted 1:100 in PBS or 200 proof Molecular Biology ethanol (PS) and allowed to evaporate at RT after >16 h of incubation at 4°C. Plates were washed 5 times with 200μL of PBS 0.05% tween 20 and then blocked for 1 h with 200μL of PBS 3% BSA. After blocking, 100μL of plasma from mice was diluted at 1:100 in blocking buffer and incubated for 2 h at 37°C. Plates were washed again 5 times and incubated with 100μL of anti-mouse IgG-HRP (GE Healthcare) diluted at 1:2000 for 1 h at 37°C. Plates were washed 5 more times and 100μL of TMB substrate (BD Biosciences) was added until desired color was obtained. Reaction was stopped by with 50μL of Stop buffer (Biolegend) and absorbance was read at 450 nm. The mean OD at 450 nm from triplicate wells representing at least 3 independent infected mice was plotted in the graphs. For human plasma ELISAs, a similar process was done, HAT patient plasma diluted 1:100 and anti-human IgM-HRP (Millipore) or anti-human IgG-HRP (GE Healthcare) for detection. The same dilution of a reference positive serum was used to calculate relative units (RU). Parasite and cell lysates were prepared in RPMI 1640 medium by 10 freeze/thaw cycles.

### ELISPOT assay

Mouse ELISPOTs were done as previously reported [12]. Mouse splenocyte suspensions from uninfected control and *T*. *b*. *brucei-*infected mice (day 10 post infection) were obtained. White blood cells were additionally purified in 45% Percoll at 25°C for 20 min at 1500 × *g* to remove remaining erythrocytes. Specific B-cell populations were enriched through magnetic bead sorting (Miltenyi) by positive selection with anti-CD11c (atypical) coated magnetic beads following manufacturer’s protocol. Purification yield was assessed by flow cytometry prior to addition to plate. 5 × 10^4^ cells were added per well and incubated in RPMI 1640 supplemented with 10% FBS in 96-well Costar 3590 ELISA plates (Corning Life Sciences, Tewksbury, MA) precoated with 100μL of either capture anti-IgG (15 mg/ml), uninfected erythrocyte lysate (10^9^ erythrocytes/ml in PBS), *T*. *b*. *brucei* or *T*. *cruzi* lysates *(*10^6^ parasites/μl in PBS) diluted 1:500 in PBS, 25 μg/ml of Calf thymus DNA (Sigma) in PBS, PS at 20 μg/ml in 200 proof Molecular Biology ethanol or PBS 10% BSA as control for 20 h at 37°C with 5% CO2. Plates were washed 5 times with 200μL of PBS 0.05% tween 20 and then it was blocked with 200μL of PBS 10% BSA for 1hr. Plates were washed 5 more times, 100μL of anti-mouse IgG biotinylated detection antibody (Sigma, St. Louis, MO) was added at 1 μg/ml diluted in PBS 0.5% FBS for 2 h at RT. 100μL of Streptavidin-horseradish peroxidase (Mabtech AB, Nacka Strand, Sweden) was added diluted in PBS 0.5% FBS for 1 h at RT. Plates were developed with 100μL of TMB substrate (Mabtech AB, Nacka Strand, Sweden) for 15–20 min and then washed extensively with water. Spots were quantified by microscopy.

### *In vitro* activation assays

Naive B cells from Swiss Webster mice were purified as a CD43 negative fraction using anti-CD43 beads (Miltenyi) and confirmed by flow cytometry to express CD19 (>96%) according to manufacturer’s protocol. Cells were cultured in B-cell medium (RPMI 1640 10% Fetal bovine serum (FBS), 100 U/mL Penicillin/Streptomycin, 2nM non-essential amino acids, 2mM L-Glutamine, 10mM Hepes and 50μl β-ME) at 5 × 10^6^ cells per ml for 2 or 3 days as indicated before analyzed by flow cytometry for T-bet expression. TLR7 agonist R848 (Mabtech) was used at 1 μg/ml; anti-BCR (Fab′)_2_ anti-IgM (The Jackson Laboratory) was used at 5 μg/ml, and IFN-γ (Peprotech) at 100 U/ml. Uninfected (3T3 mouse fibroblasts) and infected parasite lysates (*P*. *yoelii* 17XNL strain from mice-infected blood [12], *T*. *b*. *brucei* or *T*. *cruzi+/-* fibroblasts from *in-vitro* cultures as described previously [20]) were added at a concentration of 1:10 (B-cell: infected cell), as measured before lysis. Parasite and cell lysates were prepared in B-cell medium by 10 freeze/thaw cycles.

### Patients

Human patient and local control plasma samples were collected in 2002–2003 in Uganda according to protocols approved by the Grampian Research Ethics Committee (Aberdeen, UK) and the Ministry of Health (Uganda). Patients were identified after either self-presentation in a hospital setting or through community surveillance. Individuals (or their guardians) signed consent forms after receiving standard information in their local language. The samples analyzed here were a subset of those previously described originally [13](Table 1). Follow up samples were taken on completion of chemotherapy and confirmation of negative parasitology 34–60 days post recruitment. Importantly, individuals presenting with malarial co-infections were excluded from the analysis. For patients, malaria co-infection was excluded on the basis of negative blood smear. Symptomatic exclusion is not possible due to symptom spectrum overlap with HAT. For endemic controls, malaria co-infection exclusion was based by symptoms and blood smear. Full detail of co-infection exclusion approach is in the original study [13,21].

**Table 1:** (related to Fig 6). Demographics for HAT patients and endemic community controls.

	CC (n = 18)	HAT (n = 39)
Age (years)	37.5(28, 50.5)	26 (15.5, 40.5)
Female sex (%)	9(50)	27 (67.5)
District (n, %)	Serere (16, 88.8) Tororo (2, 11.1)	Busia (7, 17.9) Bugiri (2, 5.1) Kaberamaido (1, 2.6) Jinja (1, 2.6) Serere(22, 56.4) Tororo (6, 15.3)
Follow up (n)	---	19

Data presented as median (IQR) unless otherwise indicated. Sample collection as described in [13]

Abbreviations: community controls (CC), Human African Trypanosomiasis patients (HAT).

### Statistical analysis

All analyses were performed using GraphPad Prism version 5.0 (GraphPad Software, La Jolla, CA). For mice experiments: Error bars represent the standard deviation from at least 3 mice assessed independently unless otherwise stated. We quantified *P* values using *t*-student, non-parametric Spearman correlation or one-way ANOVA with Turkey’s multiple comparison test as indicated. *P <* 0.05 was considered statistically significant.

## Results

### ABCs and autoantibodies expand during *T*. *b*. *brucei*, but not *T*. *cruzi*, acute infection in mice

**Fig 1:**
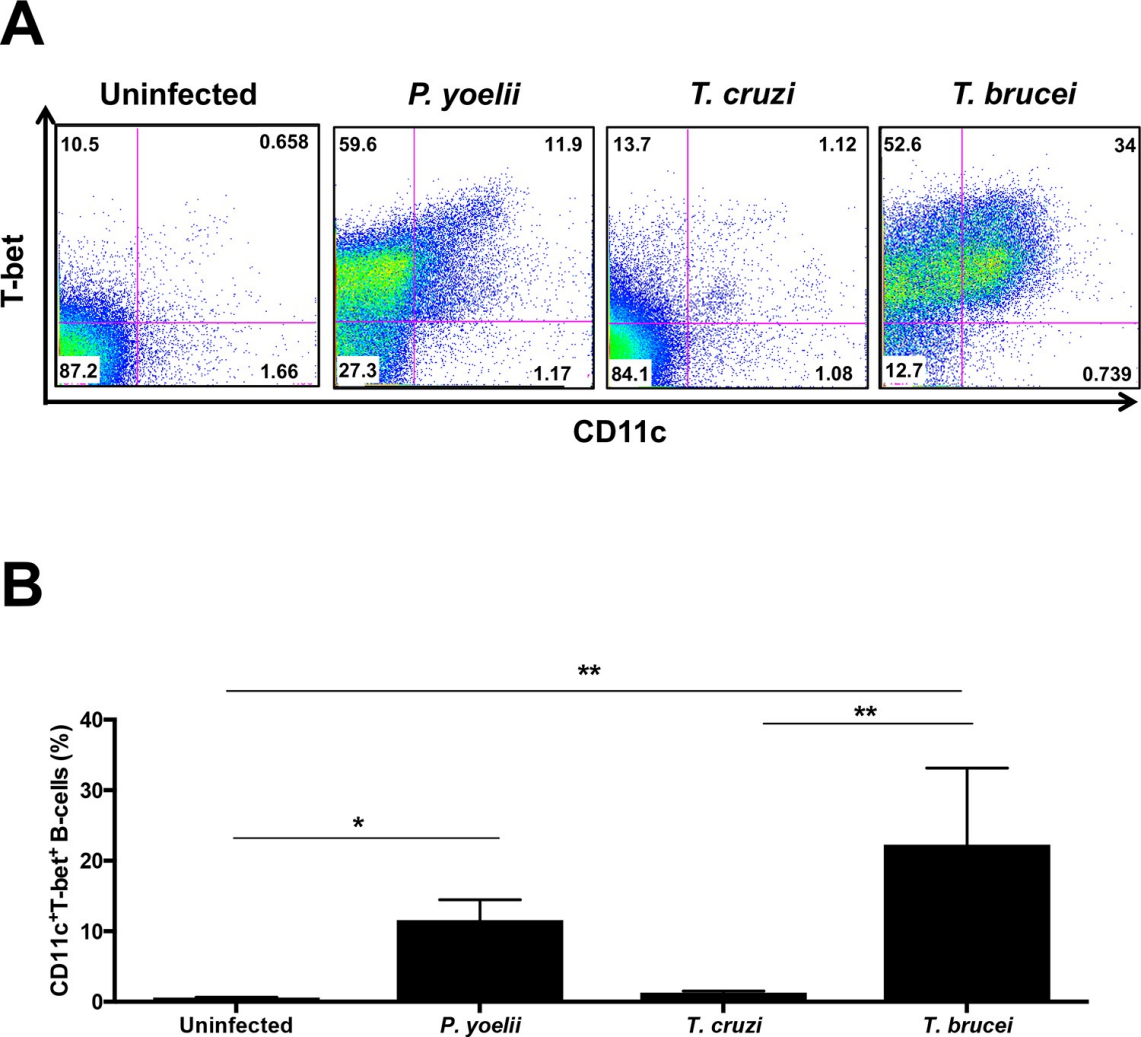
ABCs expand during acute *T*. *b*. *brucei* infection in mice. (a) Representative plots of gated CD19^+^ splenocytes to identify CD11c^+^ T-bet^+^ B cells from uninfected, day 8 post-infection with either *P*. *yoelii*, *T*. *cruzi* or *T*. *b*. *brucei*-infected mice. (b) Quantification of CD11c^+^ T-bet^+^ B cells in (a). Bar graph represents the means ± SD of n = 3 mice from at least two independent experiments. Significance assessed by one-way ANOVA with Turkey’s multiple comparison test. *p < 0.05 **p≤0.01.

We first assessed whether ABCs expanded in mouse acute infection models of *T*. *b*. *brucei* and *T*. *cruzi*. After gating out non-B cells (CD19^−^) in splenic lymphocytes (S1 Fig), we identified an ABC population, as defined by high expression of CD11c and T-bet [17,18], which expanded in *T*. *b*. *brucei-*infected mice (Fig 1). *T*. *b*. *brucei-*infected mice had significantly higher levels of ABCs compared to uninfected mice, and similar levels compared to mice infected with the mouse malaria parasite *P*. *yoelii*, which also induces the expansion of this population [12]. *T*. *cruzi*–infected mice did not significantly induce expansion of ABCs.

### Longitudinal analysis of autoantibodies and anemia during trypanosome infection in mice

**Fig 2:**
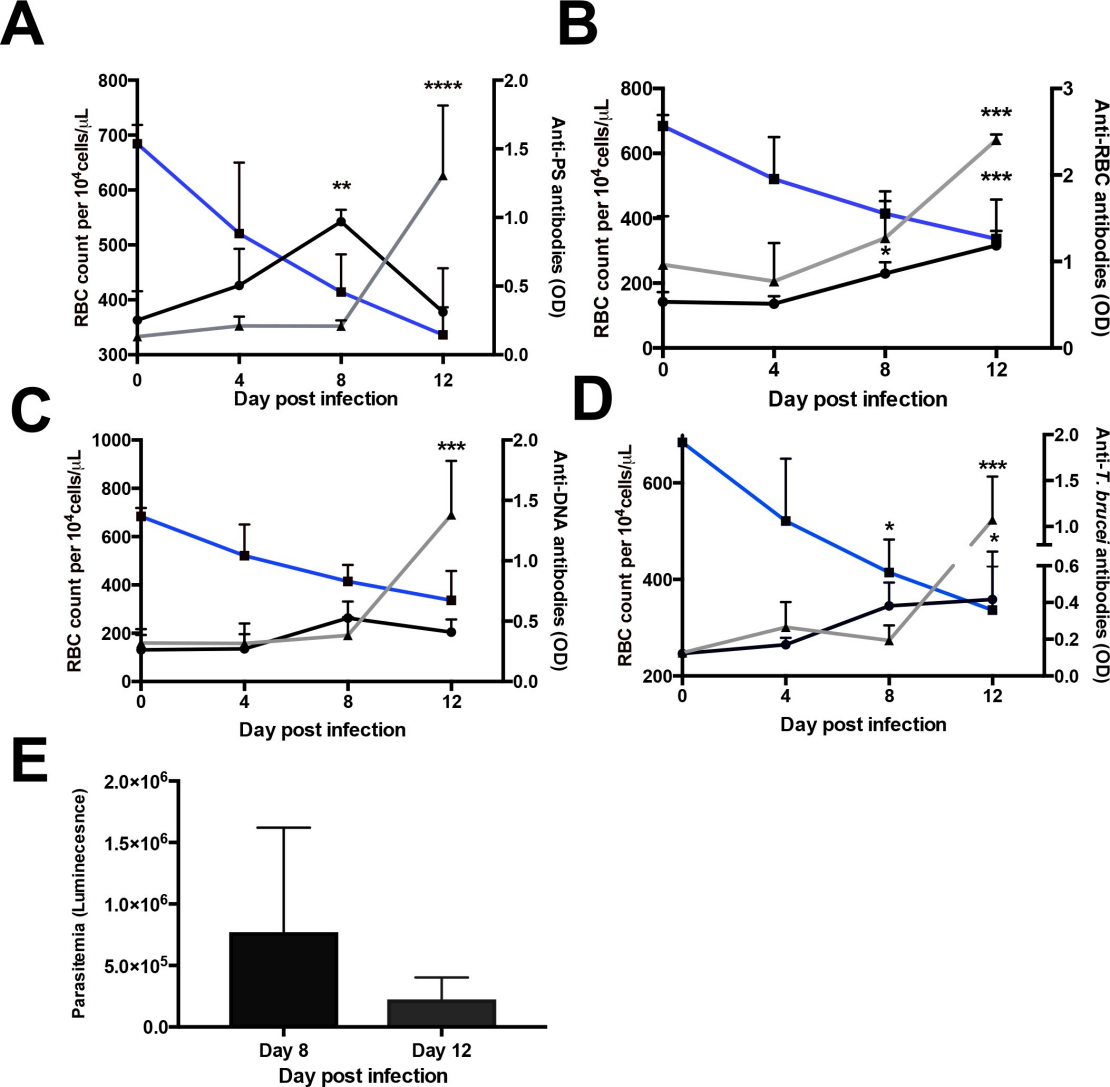
Antibodies and anemia during acute *T*. *brucei* infection in mice. Longitudinal analysis of erythrocyte count (blue line), IgM (black line) and IgG (grey line) autoantibodies (a-c) or anti-*T*. *brucei* antibodies (d) in *T*. *brucei*-infected mice. (e) Bar graph with average parasitemia of mice infected with *T*. *brucei*. Line and bar graphs represent the means ± SD of n = 3–6 mice per time point from two independent experiments. Significance between erythrocyte counts or levels of antibodies at any day post-infection compared to day 0 was determined by one-way ANOVA. *p < 0.05, **p < 0.01, ***p < 0.001, ****p < 0.0001.

**Fig 3:**
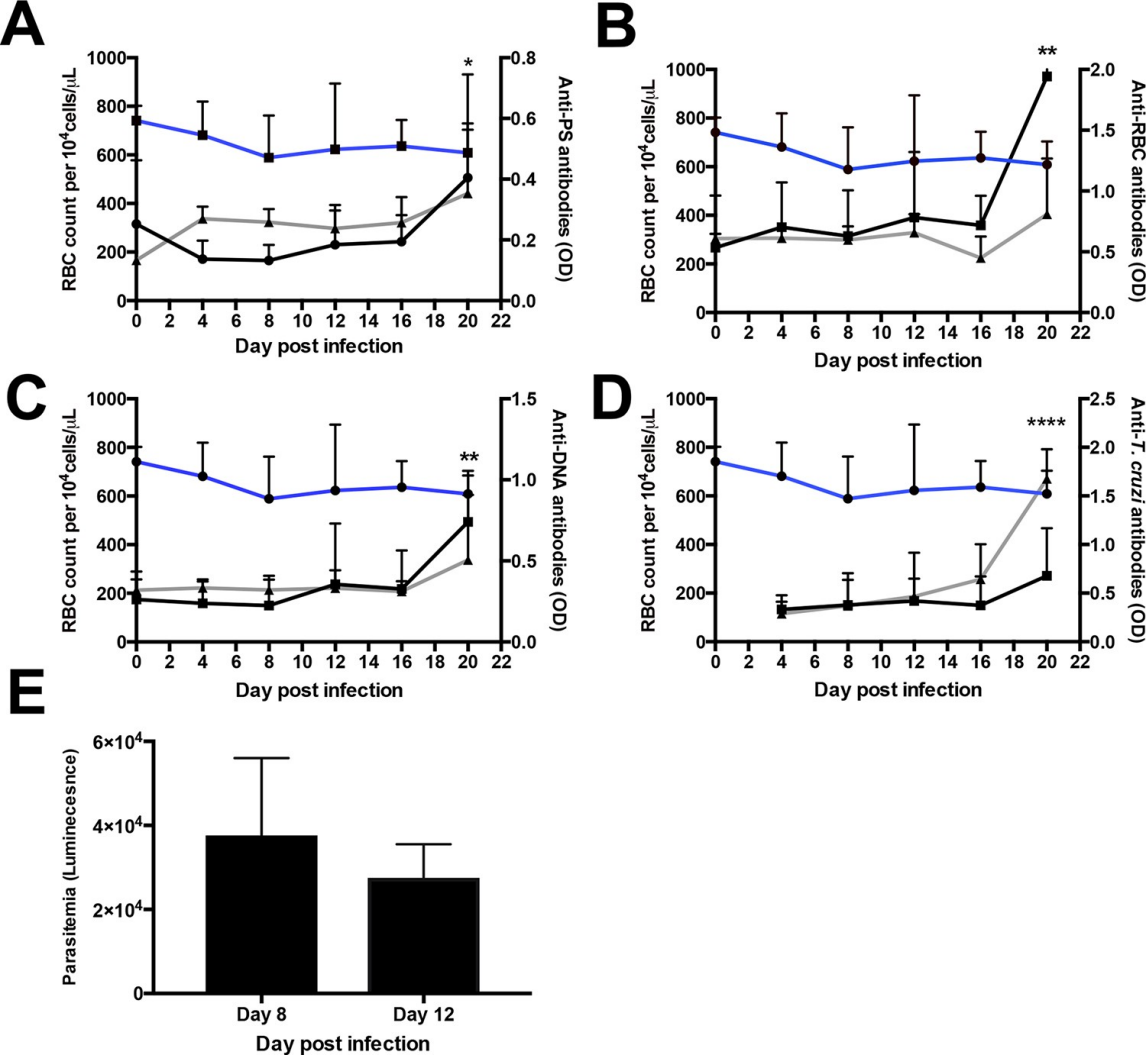
Antibodies and anemia during acute *T*. *cruzi* infection in mice. Longitudinal analysis of erythrocyte count (blue line), IgM (black line) and IgG (grey line) autoantibodies (a-c) or anti-*T*. *cruzi* IgG antibodies (d) in *T*. *cruzi-* infected mice. (e) Bar graph with average parasitemia of mice infected with *T*. *cruzi*. Line and bar graphs represent the means ± SD of n = 3–6 mice per time point from two independent experiments. Significance between erythrocyte counts or levels of antibodies at any day post-infection compared to day 4 was determined by one-way ANOVA. *p < 0.05, **p < 0.01, ****p < 0.0001.

Next, we set out to characterize the levels of autoantibodies to three different autoantigens, PS, DNA and a lysate of control uninfected red blood cells (RBC), in the plasma of *T*. *b*. *brucei* and *T*. *cruzi*–infected mice during early acute-phase infections. We also assessed antibodies to a lysate of *T*. *b*. *brucei* or *T*. *cruzi* for comparison. The levels of anti-parasite antibodies, as well as all three autoantibodies in *T*. *b*. *brucei–*infected mice showed an increase for both the IgM and IgG isotypes, although with different kinetics (Fig 2). Anti-PS IgM showed a sharp decline that coincides with an increase of anti-PS IgG at day 12 post infection. In contrast, anti*-*RBC lysate antibodies showed a steady increase throughout infection in both IgM and IgG, similarly to anti-*T*. *b*. *brucei* antibodies. In *T*. *cruzi*-infected mice significant levels of anti-*T*. *cruzi* antibodies were only detected at day 16 and autoantibodies at day 20 at the end of the acute phase of the infection (Fig 3). Parasitemia of both infections show a similar profile, declining after day 8 of infection (Figs 2E and 3E).

Since autoimmune antibodies, and in particular anti-PS, contribute to anemia in malaria [10], we further analyzed the relation between autoantibodies and erythrocyte levels. We observed opposite tendencies, with a progressive decrease of erythrocyte levels and a continuous increase in autoantibodies, where IgM appears earlier than IgG. No relationship was found between any antibody and erythrocyte levels in *T*. *cruzi*-infected mice since they did not suffer anemia (Fig 3).

### ABCs are able to secrete autoantibodies during acute *T*. *b*. *brucei* infection in mice

**Fig 4:**
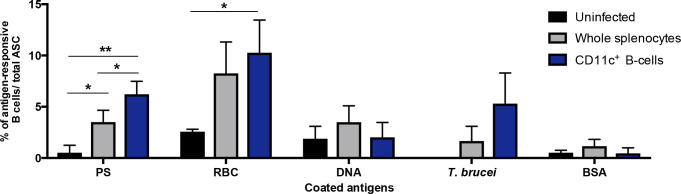
ABCs secrete autoantibodies during acute *T*. *b*. *brucei* infection in mice. B-cell ELISPOT of whole splenocytes or enriched CD11c+ B cells from uninfected and *T*. *b*. *brucei*-infected mice at day 10 post infection against coated antigens: Phosphatidylserine (PS), uninfected red blood cell lysate (RBC), Calf thymus DNA, *Trypanosoma brucei* parasite lysate and bovine serum albumin (BSA). Each data point represents the mean ± SD of n  =  3 mice. Experiments were repeated 2 times, one representative example is shown. Significance determined by one-way ANOVA. *p < 0.05, **p < 0.01.

We next wanted to assess the capacity of ABCs to secrete autoantibodies during *T*. *b*. *brucei* infection in mice by B-cell ELISPOT. Plates coated with our 3 autoantigens (PS, DNA or RBC lysate) were incubated with splenocytes from infected (day 10) or control uninfected mice. We observed the appearance of spots in wells incubated with splenocytes from infected mice, each spot representing one antibody-secreting cell recognizing the coated antigen (Fig 4). To determine whether these spots were being produced specifically by ABCs, we enriched these cells using CD11c positive selection by magnetic beads. We observed that CD11c^+^-enriched fractions, representing ABCs, had significantly higher number of antibody-secreting cells (ASCs) against PS compared to equal numbers of whole splenocytes from infected mice. All other coated antigens had no significant differences in spot numbers between total splenocytes and the CD11c^+^-enriched fraction.

### *T*. *b*. *brucei* lysates along with IFN-γ induce differentiation of ABCs *in vitro*

To understand the mechanism by which ABCs expand during *T*. *b*. *brucei* infection, we studied the effect that *T*. *b*. *brucei* antigens can have in directly promoting ABC differentiation. It is well characterized that differentiation of ABCs from naïve B-cells is promoted by the cytokine IFN-γ, which induces the characteristic expression of T-bet in these cells [19]. Since IFN-γ is found in high concentrations in the inflammatory environment induced by acute *T*. *brucei* infection [22], it is likely that B-cells are exposed to this cytokine during infection.

**Fig 5:**
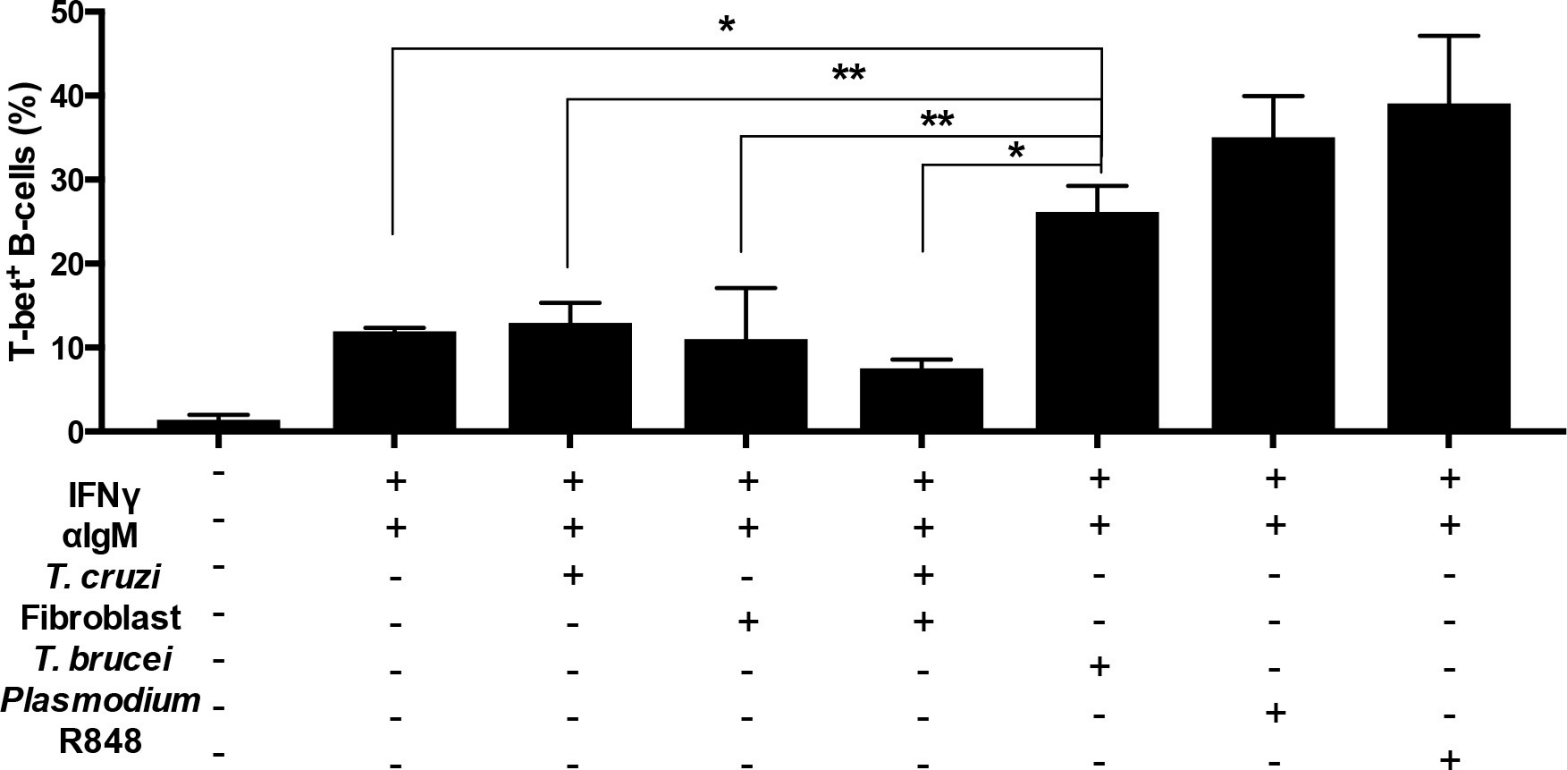
IFNγ synergizes with trypanosome molecules to induce T-bet expression on B cells *in-vitro*. Purified naive B cells (CD43−) from uninfected mice were cultured under the indicated conditions for 3 days, when T-bet expression was determined in CD19+ cells. Agonist of TLR7 (R848). Experiments were repeated 2 times, one representative example is shown. Bars represent the means ± SD of n  =  3 mice. Significance determined by one-way ANOVA. *p < 0.05, **p < 0.01.

To determine whether trypanosome molecules can induce the expression of T-bet on B cells, we isolated naive B cells from control uninfected mice and incubated them *in vitro* with lysates of *T*. *b*. *brucei* blood forms or a lysate of *T*. *cruzi*-infected fibroblasts or trypomastigotes, alone or in combination with IFN-γ. As positive control, a lysate of *P*. *yoelii* infected erythrocytes and the TLR-7 agonist R848 [12] were used to stimulate cells. Uninfected fibroblasts lysates were used as negative controls. B-cell receptor crosslinking (anti-IgM) was added to bypass the need for antigen specificity. We observed that addition of each of the three signals (IFN-γ, anti-IgM, and respective lysate) independently induces a very minor increase in T-bet expression in B cells. Combinations of these signals two-by-two, and when the three were added together, results in a synergistic increase in T-bet^+^ B cells (Fig 5). Particularly, the three signals including *T*. *b*. *brucei* lysate had a strong T-bet induction comparable to the positive control combinations. The combinations of three signals using *T*. *cruzi*-infected fibroblasts, trypomastigotes or uninfected fibroblasts lysates did not augment T-bet induction more than the two signals IFN-γ and anti-IgM. Taken together, these results indicate that *T*. *b*. *brucei* molecules can contribute to the differentiation of T-bet expressing ABCs.

### Blocking of PS during *T*. *b*. *brucei* infection in mice accelerates recovery from anemia

**Fig 6:**
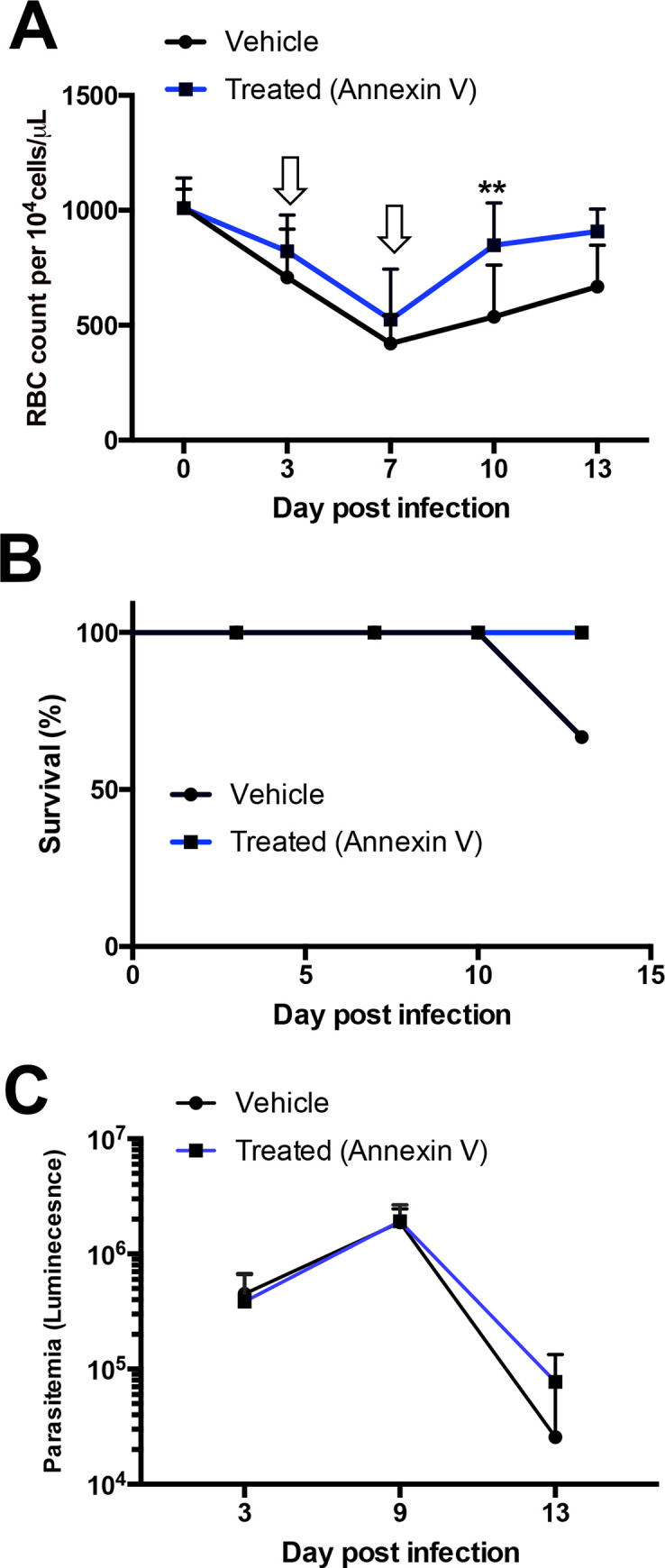
Annexin V ameliorates anemia during *T*. *b*. *brucei* infection in mice. (a-d) *T*. *b*. *brucei*-infected groups of mice (n = 3 for each condition) were injected with annexin V (blue line) or vehicle alone (black line) at days 3 and 7 post infection. Number of erythrocytes per volume (a), survival (b) and average of parasitemia (c) of mice groups were measured over time. Line graphs represent the means ± SD of n = 6 mice per time point from two independent experiments. Significance determined by unpaired student T-test. *p < 0.05, **p < 0.01.

To study the role of PS exposure in *T*. *brucei*-induced anemia, *T*. *b*. *brucei-*infected mice were injected with annexin V, which specifically binds to PS exposed on cell membranes preventing its interaction with other molecules [23,24]. We observed a significantly faster recovery from anemia in annexin V-treated mice compared to vehicle-treated mice (Fig 6A). All annexin V-treated mice survived at day 13 post-infection compared to 66.6% (4/6 mice) of the vehicle group (Fig 6B). The parasitemia of both groups of mice was similar (Fig 6C), indicating that the differences in erythrocyte levels and survival were not caused directly by the parasite. These results suggest that blocking of PS by annexin V binding could inhibit erythrophagocytosis and accelerate anemia recovery during infection.

### Anti-PS antibodies are elevated in Human African Trypanosomiasis patients

**Fig 7:**
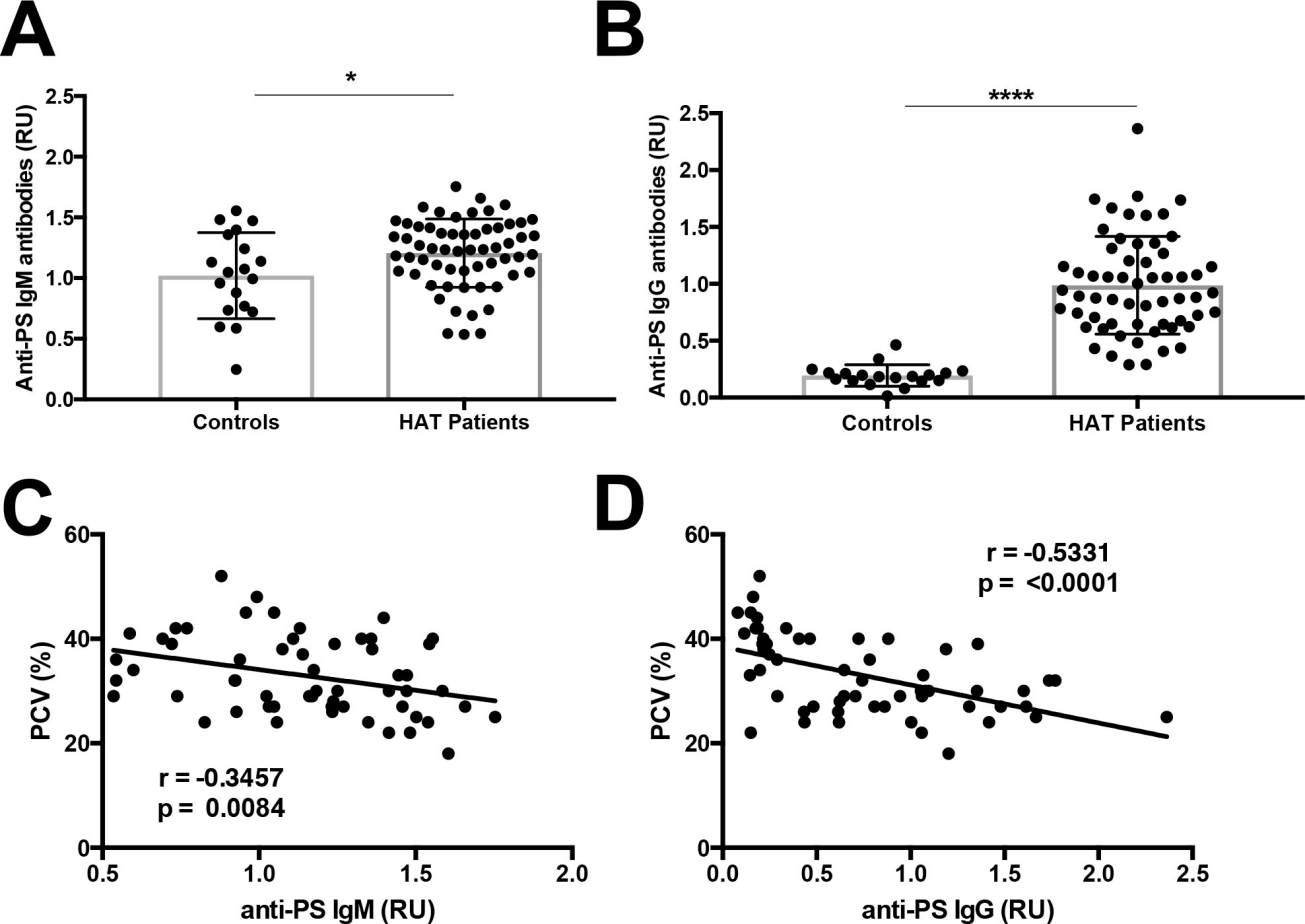
Human African Trypanosomiasis induces a strong anti-PS antibody response that is inversely correlated with erythrocyte levels. Levels of anti-PS IgM (a) or IgG (b) antibodies from HAT patients (n = 58 unique samples) or HAT-negative endemic controls (n = 18). Non-parametric Spearman correlation analysis of anti-PS IgM (c) or IgG (d) with packed cell volume (PCV) of HAT patients at initial sample (n = 39) and HAT-negative endemic controls (n = 18). Significance assessed by unpaired student T-test or Spearman correlation analysis. *p < 0.05, ****p < 0.0001.

To study whether anti-PS antibodies are present in Human African Trypanosomiasis (HAT) patients, we analyzed the plasma of HAT patients from South East Uganda infected with *T*. *brucei rhodesiense* along with uninfected local control donors (Table 1). We observed that HAT patients present higher levels of IgM, but more significantly of IgG, anti-PS antibodies compared to endemic controls (Fig 7A and 7B).

To determine whether these increases in anti-PS antibodies correlate with anemia in HAT patients, we assessed the relationship between anti-PS antibodies and hematocrit, measured as packed cell volume (PCV). We found a significant negative correlation of PCV levels in HAT patients with anti-PS IgM, but more strongly with anti-PS IgG (Fig 7C and 7D). We found no significant differences in the levels of anti-PS antibodies comparing samples taken during acute disease with follow up samples from HAT patients, (S2 Fig). However, the levels of anti-PS antibodies, both IgM and IgG, remain significantly different from healthy controls (p = 0.0394 and 0.0001, respectively) in follow up samples, indicating that anti-PS remain elevated after acute disease for at least one to two months. There was no correlation between PCV and anti-PS antibodies in endemic non-HAT controls (S2D and S2E Fig). Taken together, our results indicate that, similarly to mice, *T*. *brucei rhodesiense* human infection also induces the generation of anti-PS autoantibodies that could contribute to anemia in humans.

## Discussion

Anemia during infection with trypanosomes is a commonly reported complication in wide range of hosts [25], but remains incompletely understood. Autoimmunity has been suggested to play an important role in promoting pathology during trypanosome infection [3,26]. The objective of this study was to assess whether autoimmunity against PS by atypical B-cells had a role in acute anemia during infection with trypanosomes, hence expanding our previous studies of acute anemia during malaria [7–12]. During malaria, autoimmunity against PS is a transient phenomenon that contributes to acute-phase anemia through clearance of RBCs mediated by the binding of anti-PS autoantibodies to exposed PS on RBC [10]. However, the role that autoimmune antibodies play during acute-phase anemia in *T*. *brucei* infection has not been thoroughly explored. To our knowledge this is the first study to assess the levels of ABCs, autoantibodies, and their role in anemia development during acute *T*. *brucei* infection in mice.

ABCs were first discovered in aged mice and mouse models of autoimmunity, and have been the hallmark of different human autoimmune diseases [17,18]. Our previous work has established a key role for ABCs in promoting malarial anemia, through the secretion of anti-PS and other autoantibodies, in both mice and patients [8,9,11,12]. These studies raised the question whether ABCs could be playing a similar role during other highly inflammatory infections.

Increases in specific autoantibodies, particularly anti-PS IgG, are observed during early *T*. *b*. *brucei*-infection in mice at the time of ABC expansion. The differentiation of ABCs during autoimmunity has been described to depend on three different signals: B-cell receptor signaling, Type 1 inflammatory cytokines such as IFN-γ and IL21 and a nucleic acid receptor such as TLR7 or 9 [17,18]. During malarial infection, parasite DNA sensing through TLR9 along with IFN-γ had a prominent role in ABC expansion [12]. We found here that *T*. *b*. *brucei* molecules, coming from a whole parasite lysate, can also synergize with IFN-γ to promote ABC differentiation *in-vitro* as measured by T-bet induction, suggesting that *T*. *b*. *brucei* RNA or DNA may contribute to this process.

Comparing *T*. *b*. *brucei* and *T*. *cruzi* acute infections in mice, we observed similar parasitemia kinetics, however the anti-PS response was quite different since *T*. *cruzi* infection induced no detectable anti-PS until day 20, while *T*. *brucei* infected mice show significant titers at day 8 for IgM and day 12 for IgG. Our results also show that acute *T*. *b*. *brucei*, but not *T*. *cruzi*, infection in mice led to robust expansion of ABCs that are able to secrete autoantibodies, particularly anti-PS IgG, during infection in mice. Since *T*. *b*. *brucei*, but not *T*. *cruzi*, lysates were able to induce the expansion of ABCs *in vitro* it is likely that the difference in ABC activation and autoimmune antibody levels during the two infections may be mediated by the intrinsic capacity of *T*. *b*. *brucei* molecules to trigger this response.

Anemia during any infection, including during *T*. *brucei* infection, is a multi-factorial pathology that involves the disruption of multiple physiological and immunological processes including erythropoiesis, erythrophagocytosis, cell lysis, iron metabolism, oxidative stress and inflammation [24,27–31]. We found that acute *T*. *cruzi* infection did not cause anemia, and did also not induce significant ABC or autoantibody levels in mice during the early acute phase of infection. These differences with *T*. *brucei* infection could be due to the divergent host immune response to these two parasitic infections, which in *T*. *brucei*-infected mice includes very high levels of inflammatory cytokines [32], extracellular vesicles [31], activation of the mononuclear phagocyte system [33], among others. Importantly, trypanosome parasites are well known to establishing chronic infections [34], hence inducing prolonged or delayed anemia [25]. The factors that contribute to chronic anemia [35] probably differ, such as iron availability [36], from the ones that are transiently present during acute-phase anemia, such as autoimmunity [11]. It remains to be determined whether anti-PS antibodies and other autoantibodies could have a role in chronic anemia by trypanosomes.

During malaria, anti-PS IgG antibodies promote anemia by binding to PS on uninfected erythrocytes and enhancing their premature clearance. *T*. *b*. *brucei* infection also leads to enhanced exposure levels of PS on erythrocytes [37] and elevated levels of anti-PS, suggesting that this mechanism could contribute to anemia.

Different studies have attributed induction of anemia during *T*. *brucei* infection to various mechanisms including hemodilution [37], impaired erythropoiesis or release of RBCs into the circulation [38] and accelerated erythrophagocytosis [39]. Indeed, activation of the mononuclear phagocyte system [33] has been attributed an essential role in mediating anemia during *T*. *brucei* infection. On the other hand, B-cells have been attributed a non-essential role in anemia induction since anemia progresses similarly in B-cell deficient mice during *T*. *brucei* infection [40]. Rather than anti-PS antibodies having a role in inducing anemia, we hypothesize that they exert their effect by delaying the recovery of RBC levels. Anti-PS binding to PS, that is largely exposed on young reticulocytes [10,41], would promote their premature clearance by phagocytes, prolonguing anemia. Accordingly, our results showed that masking PS with Annexin V accelerates RBC recovery. This enhancement in RBC recovery by Annexin V could be mediated by masking PS from phagocytes that recognize it through PS receptors or through Fc receptors recognizing anti-PS antibodies. However, our studies did not measure directly the role of anti-PS antibodies in mice and further studies that would support this hypothesis should address this point. Such experiments could include injection of purified anti-PS IgG antibodies to *T*. *brucei*-infected mice in combination or not with Annexin V and assess its effect on circulating RBC/ reticulocyte levels as well as on RBC progeny in the spleen and bone marrow. Additionally, the use of RBCs labeled with biotin or radioactive isotopes has been historically used to assess RBC recovery and survival [42]. An alternative approach could be to inject labeled RBCs into *T*. *brucei*-infected mice with a follow up challenge injection of purified anti-PS antibodies in combination +/- Annexin V and measure RBC turnover and recovery from anemia. Altogether, our data adds PS exposure and, possibly its targeting by anti-PS antibodies, as an important factor delaying RBC recovery and prolonging anemia.

Several *T*. *brucei* proteins, such as variant surface glycoprotein [43] and trypanosome lytic factor 2 [44] contain antigens that are recognized by autoimmune antibodies in mice and humans never exposed to this pathogen. *Trypanosoma brucei* infections lead to B-cell polyclonal activation [24,45] that could be a main driver causing secretion of autoantibodies. Apart from autoantibodies, other unrelated antibodies such as antibodies against irrelevant VSGs [46], have also been reported during *T*. *brucei* infection. It has been hypothesized that these irrelevant antibodies serve as way to dilute out the pathogen specific antibody responses, serving as an immune evasion mechanism and prolonging infection [47]. Our results suggest an additional interpretation where autoantibodies from this polyclonal response could also be considered damaging for the host by delaying the recovery from anemia.

The relevance of anti-PS IgG antibodies in promoting anemia has been studied in various cohorts of human malaria caused by different *Plasmodium* species [7–10,48]. Here we also report the presence of high levels of anti-PS IgM and IgG antibodies in HAT patients that correlated negatively with PCV levels, suggesting a relation between autoimmunity to PS and anemia during human acute infection.

Altogether these data suggest that autoimmune B-cells could have a role in anemia, particularly in delaying RBC recovery, through the secretion of autoantibodies during *T*. *brucei* acute infection.

## Supporting information

S1 Fig (related to Fig 1). **Gating strategy for spleen Atypical B-cells (ABCs).** Gating strategy for the characterization of CD11c+ T-bet+ B-cells (within CD19+) with representative plots of one uninfected control and one *T*. *brucei*–infected mice.
(TIF)

S2 Fig (related to Fig 7). **Follow-up parameters of HAT patients.** Follow-up analysis of anti-PS IgM (a), anti-PS IgG (b) or PCV (c) levels of HAT patient samples (n = 19) before treatment (BT) or after treatment (AT). Non-parametric Spearman correlation analysis of anti-PS IgM (d) or IgG (e) with packed cell volume (PCV) of HAT-negative endemic controls (n = 18). Significance assessed by unpaired student T-test analysis or Spearman correlation analysis. **p < 0.01.
(TIF)

S1 Data OD values for Fig 2 ELISAs. (XLSX)

S2 Data OD values for Fig 3 ELISAs. (XLSX)

S3 Data OD values for Fig 7 ELISAs. (XLSX)
