# Supplementary figures and images for: Autoimmunity to phosphatidylserine and anemia in African Trypanosome infections
Source: PLoS Negl Trop Dis. 2021 Sep 29;15(9):e0009814. doi: 10.1371/journal.pntd.0009814 (PMC8505006; doi:10.1371/journal.pntd.0009814)

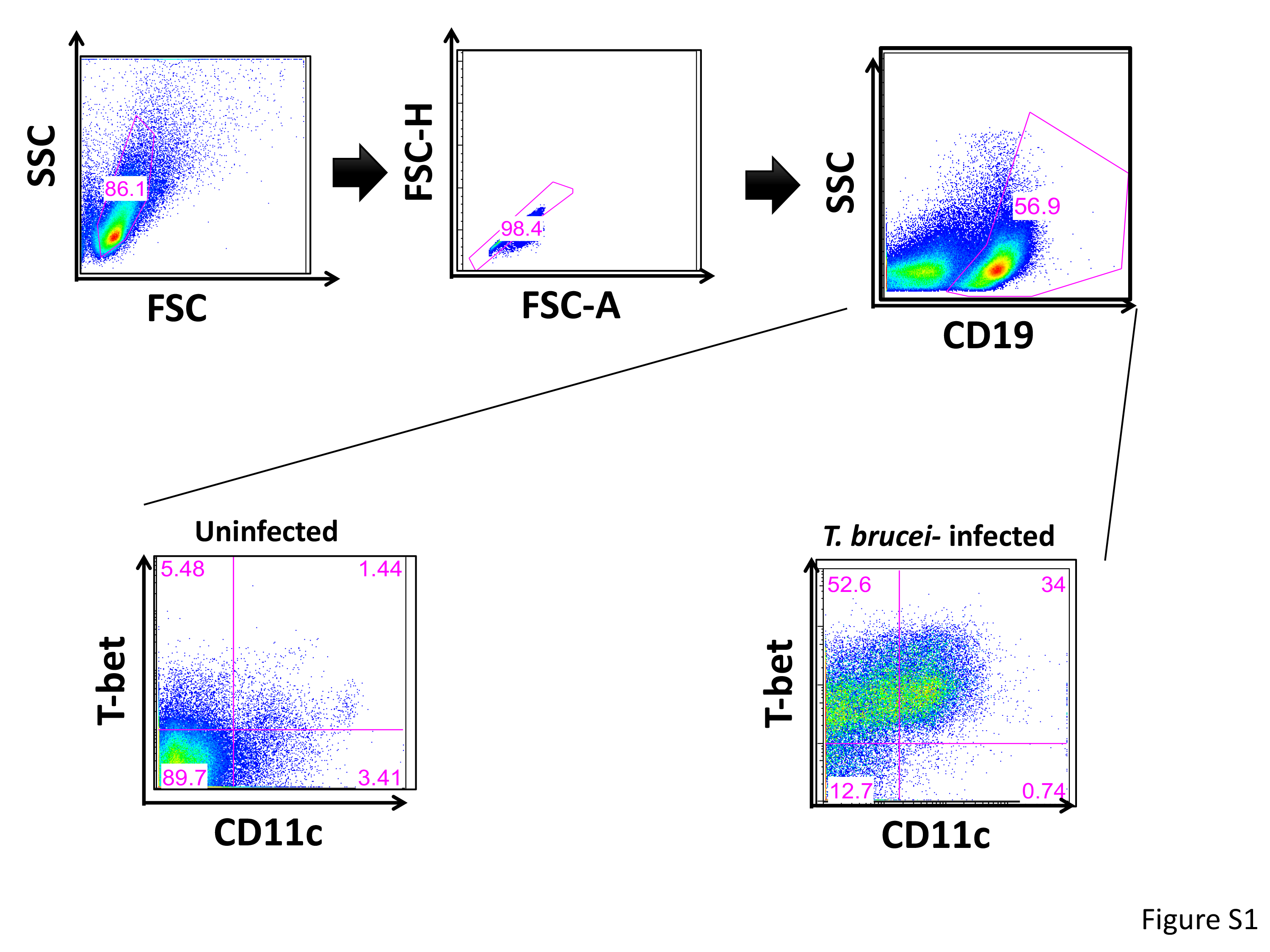

Supplement: S1 Fig — Gating strategy for spleen Atypical B-cells (ABCs). Gating strategy for the characterization of CD11c+ T-bet+ B-cells (within CD19+) with representative plots of one uninfected control and one T. brucei–infected mice. (TIF) [file pntd.0009814.s001.tif]

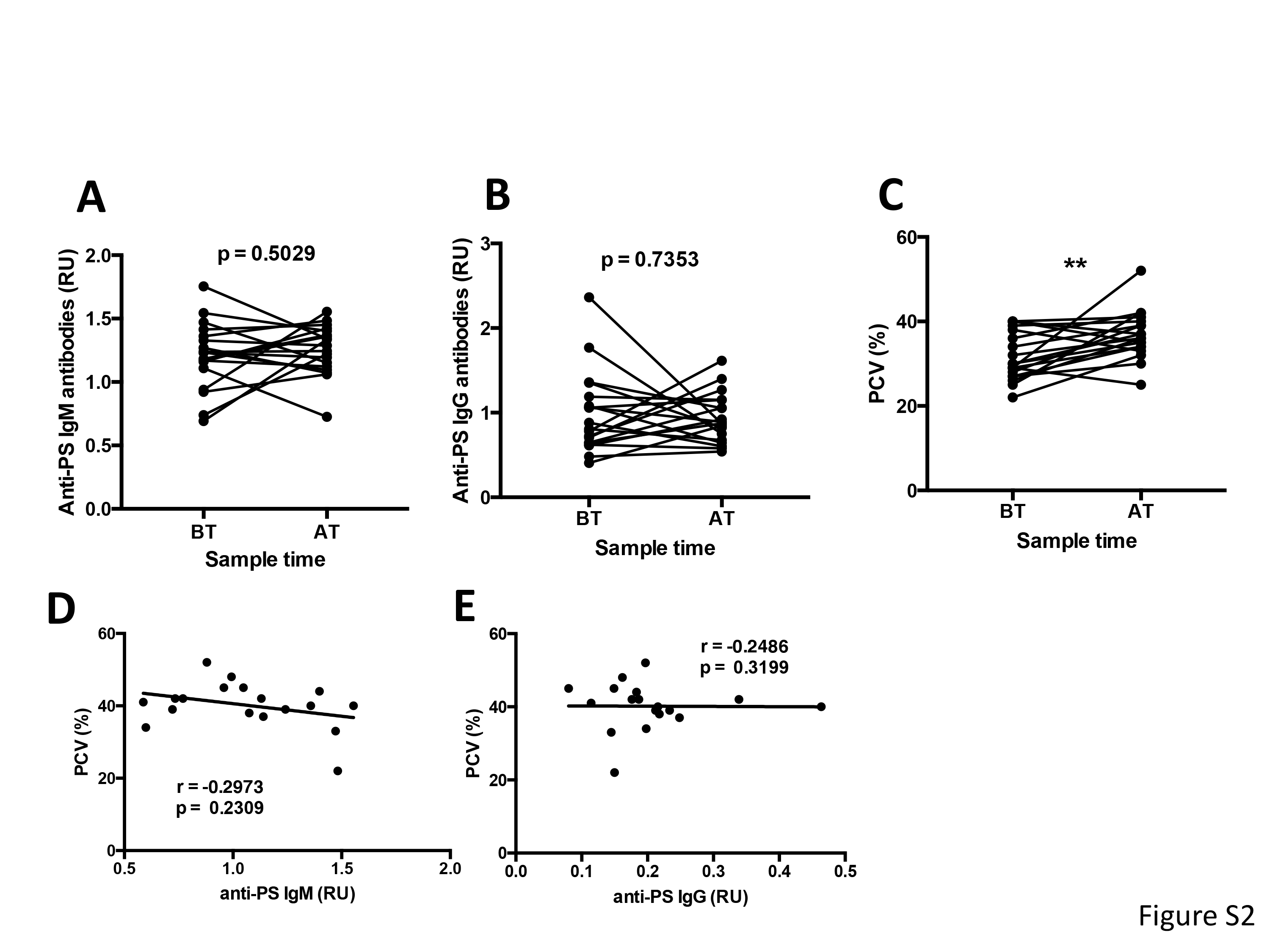

Supplement: S2 Fig — Follow-up parameters of HAT patients. Follow-up analysis of anti-PS IgM (a), anti-PS IgG (b) or PCV (c) levels of HAT patient samples (n = 19) before treatment (BT) or after treatment (AT). Non-parametric Spearman correlation analysis of anti-PS IgM (d) or IgG (e) with packed cell volume (PCV) of HAT-negative endemic controls (n = 18). Significance assessed by unpaired student T-test analysis or Spearman correlation analysis. **p < 0.01. (TIF) [file pntd.0009814.s002.tif]
